# Exploring the Rare Hobnail Variant of Papillary Thyroid Carcinoma: A Case Report

**DOI:** 10.7759/cureus.86323

**Published:** 2025-06-18

**Authors:** Fatima Zahra Bouayed, Soufiane Berhili, Mohammed El-Masadi, Mohammed El Magroud, Ahmed BenSghier, Mohamed Moukhlissi, Loubna Mezouar

**Affiliations:** 1 Radiation Oncology, Faculty of Medicine and Pharmacy, Mohammed First University, Oujda, MAR; 2 Pathology, Faculty of Medicine and Pharmacy, Mohammed First University, Oujda, MAR; 3 Radiation Oncology, Centre Hospitalier Universitaire Mohammed VI, Oujda, MAR; 4 Radiotherapy, Mohammed VI University Hospital, Oujda, MAR

**Keywords:** hobnail, palliative radiation therapy, papillary carcinoma of thyroid, radiation and clinical oncology, thyroid pathology

## Abstract

The hobnail variant of papillary thyroid carcinoma (HPTC) is a rare but highly aggressive form of thyroid cancer, associated with a more severe clinical course and significantly poorer prognosis compared to conventional PTC. To be classified as HPTC, a tumor must contain at least 30% of cells exhibiting hobnail-micropapillary features. These cells are characterized by distinctive papillary cytomorphology, including a hobnail-like appearance, the presence of papillae and micropapillae, high nuclear-to-cytoplasmic ratios, apically located nuclei, and prominent nucleoli. Additionally, some studies have reported associations between HPTC and specific genetic mutations, such as BRAFV600E, TP53, and TERT. In this article, we present the case of a 58-year-old male diagnosed with HPTC, managed with radiotherapy.

## Introduction

Thyroid cancer is the most prevalent endocrine malignancy, particularly among women, and its incidence has increased due to the widespread use of ultrasound for detection [1]. Papillary thyroid carcinoma (PTC) is the most commonly diagnosed subtype and is generally associated with favorable long-term outcomes; most patients survive for more than ten years following surgery [2]. However, certain pathological variants of PTC, such as the tall cell, columnar cell, and hobnail variants, are notably more aggressive. These subtypes tend to exhibit unfavorable clinical and pathological features, including rapid tumor progression and extensive metastatic spread at diagnosis. Moreover, they often respond poorly to conventional therapies, leading to worse prognoses [3].

Recently, the hobnail variant of PTC (HPTC) has been recognized as a rare and highly aggressive form, defined by the presence of more than 30% of tumor cells displaying hobnail-like features. These cells are characterized by a high nuclear-to-cytoplasmic ratio, apically located nuclei, and a loss of cellular polarity [4]. HPTC is associated with a reduced likelihood of cure due to its resistance to radioactive iodine therapy, a greater propensity for metastasis, and lower overall survival rates [2].

In patients presenting with concerning symptoms, radiotherapy plays an important role as a palliative treatment and should be considered in the overall management plan. Palliative radiotherapy often employs short hypofractionated regimens designed to reduce treatment duration while effectively managing symptoms. In cases of advanced-stage HPTC or when symptoms such as airway obstruction or pain are present, radiotherapy can help reduce tumor burden, alleviate symptoms, and improve quality of life.

In this article, we present the case of a 58-year-old male diagnosed with HPTC, who was managed with radiotherapy.

## Case presentation

A 58-year-old male with no family history of thyroid cancer or other malignancies presented to the Department of Radiation Oncology following the discovery of a thyroid mass that had been growing for over three months. The patient reported experiencing difficulty breathing, particularly at night. On physical examination, a hard, non-tender mass was palpable on the left side of the neck, measuring approximately 8-9 cm in diameter; the size of the mass prevented the palpation of any cervical lymph nodes. Blood tests revealed normal levels of thyroid-stimulating hormone (2.69 µUI/mL), with elevated levels of thyroglobulin (343.80 ng/mL) and anti-thyroglobulin antibodies (2056.00 U/L) (Table 1).

**Table 1:** Comparison of the patient’s biological data with standard reference values

Parameter	Patient value	Reference range
Thyroid-stimulating hormone	2.69 µUI/mL	0.30-4.85 µUI/mL
Thyroglobulin	343.80 ng/mL	3.5-77.00 ng/mL
Anti-thyroglobulin antibodies	2056.00 U/L	<115.00 U/L

**Figure 1:**
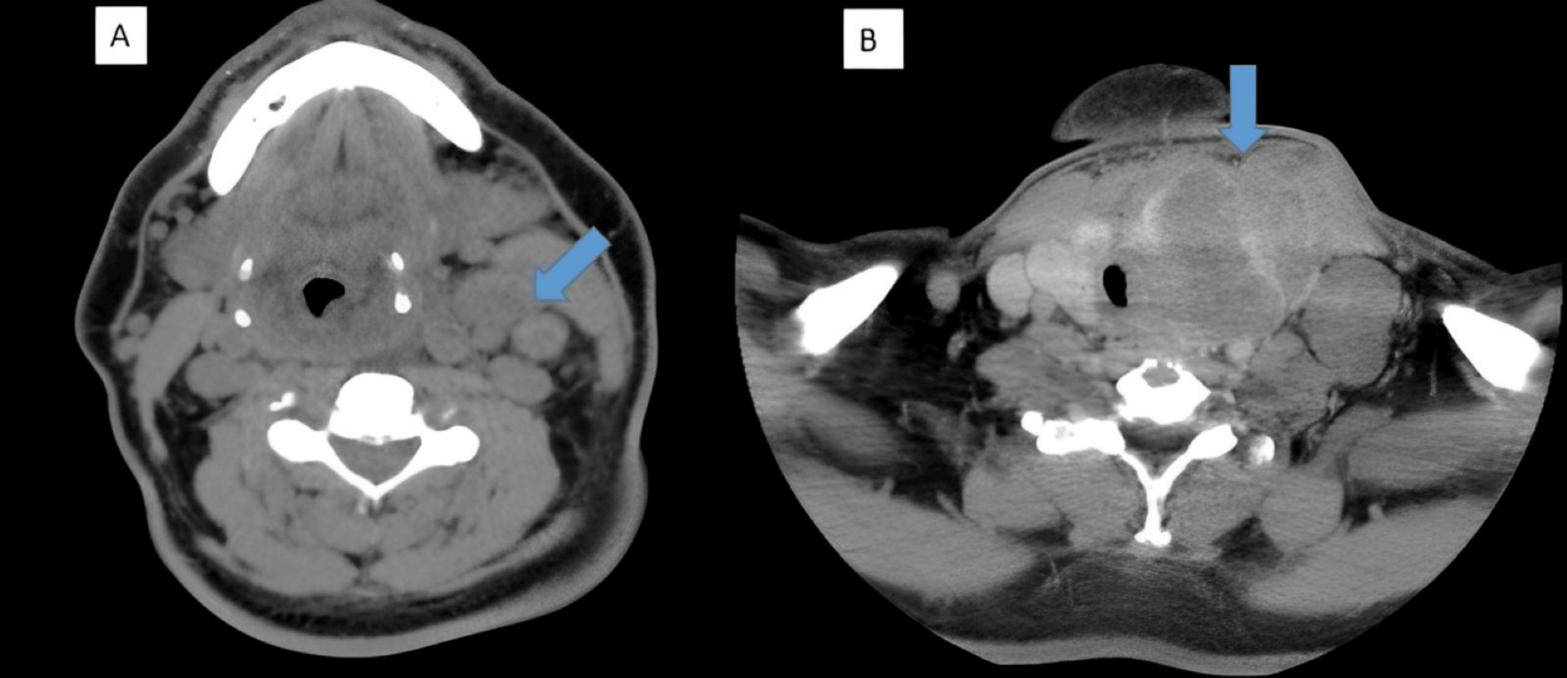
Cervical-thoracic CT (A) Axial section showing left jugulo-carotid adenopathy. (B) Axial section revealing a large tumor in the left thyroid lobe, extending into the thyroid isthmus and invading the left sternocleidomastoid muscle anteriorly and toward the right. There is also posterior vertebral invasion with lytic destruction of the C7 vertebral body. This mass causes medial displacement and compression of the trachea, along with lateral compression of the left jugular vein. Multiple left supraclavicular lymphadenopathies are also observed.

The initial thyroid ultrasound revealed left isthmic-lobar hypertrophy with a heterogeneous multinodular appearance, classified as EU-TIRADS 4. Fine-needle aspiration was performed and showed morphological features consistent with the HPTC. Cervical-thoracic CT revealed a voluminous tumor in the left isthmic lobe of the thyroid, extending into the right lobe, measuring 90 × 75 × 61 mm at its largest dimensions. The tumor demonstrated extensive local invasion: it infiltrated the left side of the larynx, causing lysis of the cricoid cartilage and condensation of the left arytenoid cartilage, as well as involvement of the left lateral tracheal wall. It encased the left esophageal wall over a length of more than 45 mm and extensively infiltrated the left sternocleidomastoid muscle. The mass displaced and compressed the internal carotid-jugular vascular bundle, resulting in a laminated appearance of the internal jugular vein. It also extended into the prevertebral space, with evidence of minor osteolysis of the anterior vertebral body of C7. A cluster of enlarged left cervical lymph nodes was observed, with the largest measuring 25 × 15 × 23 mm (Figure 1).

**Figure 2:**
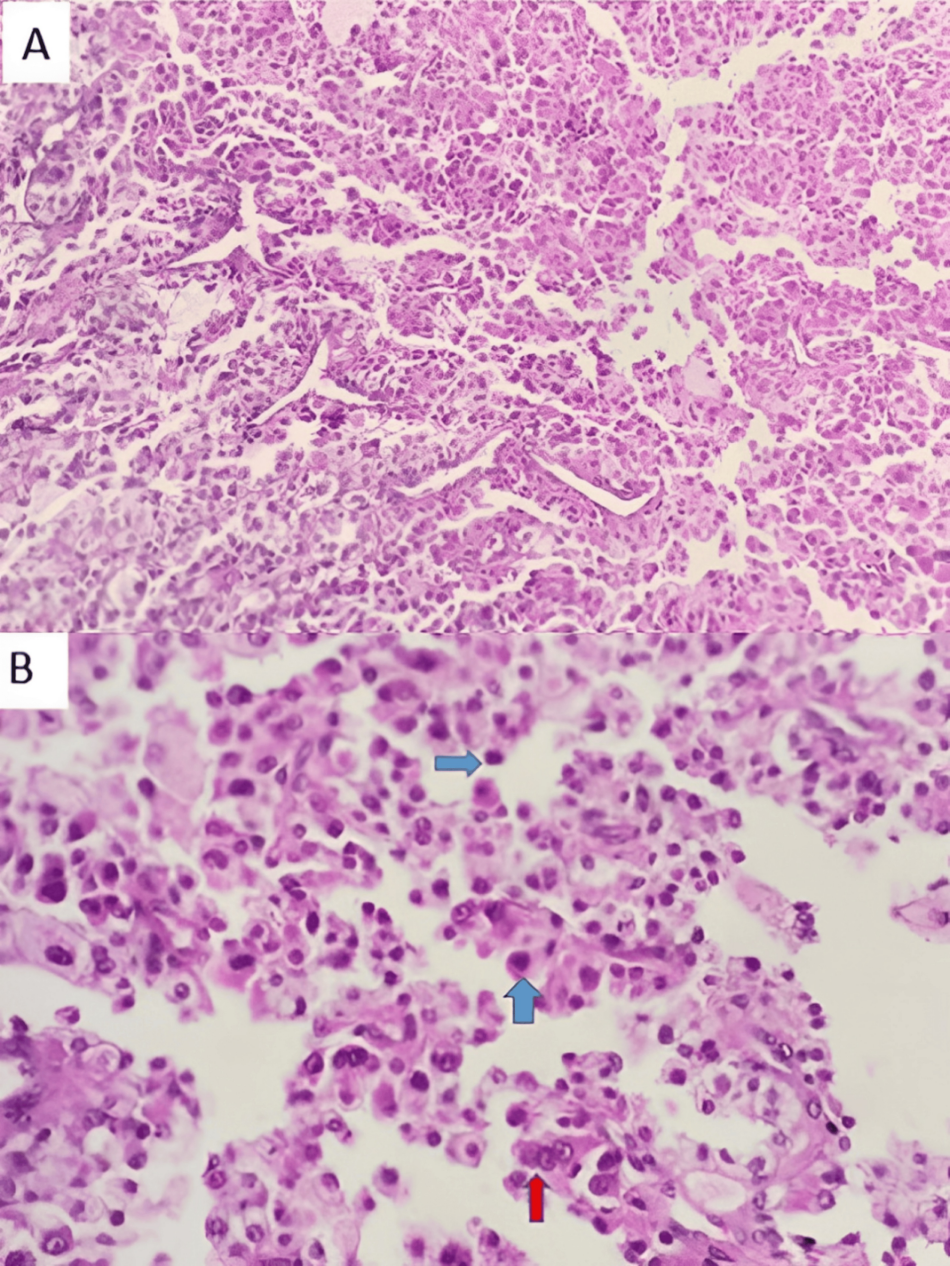
Histopathological analysis of the thyroid fine needle biopsy (A) Carcinomatous proliferation displaying a papillary architecture (H&E, ×100). (B) At higher magnification, the tumor cells exhibit characteristic nuclear features of PTC, including nuclear enlargement, overlapping (red arrows), chromatin clearing and margination, nuclear grooves, and pseudo-inclusions. The cells also demonstrate hobnailing (blue arrows) and reversed polarity, with nuclei protruding into the lumen (H&E, ×200). PTC, papillary thyroid carcinoma

Pathological examination revealed a carcinomatous tumor proliferation composed of papillae, micropapillae, and diffuse sheets of large cells exhibiting nuclei with inverted polarity and marked anisokaryosis, along with dense chromatin and prominent nucleoli. The tumor stroma was fibrotic and variably distributed, containing scattered inflammatory cells. The tumor infiltrated the surrounding muscle tissue, and multiple intravascular tumor emboli were identified (Figure 2). These findings were morphologically consistent with a diagnosis of HPTC. 

A cervical-thoracic-abdominal-pelvic CT scan was performed, and no evidence of metastatic spread was found. The case was discussed in a multidisciplinary tumor board, where surgery was initially suggested as the treatment of choice. However, due to the extensive burden of the disease, the patient was deemed unsuitable for surgery and was referred to our department for decompressive radiotherapy, aimed at reducing the risk of suffocation or other respiratory complications.

**Figure 3:**
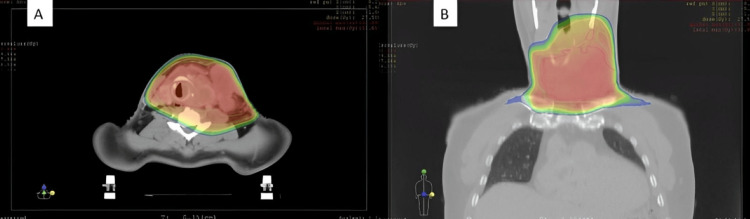
Dosimetric evaluation of radiotherapy: colorwash dose coverage of the primary tumor bed (A) Axial section. (B) Coronal section.

The patient underwent external three-dimensional conformal radiotherapy targeting the tumor, receiving a total dose of 30 Gy, delivered in 10 fractions of 3 Gy each (Figure 3). The patient experienced mild grade 1 radiation dermatitis, which was easily managed with symptomatic treatment.

Curative radioiodine treatment was also considered not safely feasible due to the extent of tumor expansion and the presence of remaining intact thyroid tissue. For six months following radiation therapy, the patient remained clinically stable, with notable improvement in breathing difficulties, particularly nocturnal dyspnea, and no evidence of a compressive cervical mass or distant metastases.

However, one month later, the patient suffered a sudden cerebral hemorrhage caused by poorly controlled hypertension. He was admitted to the ICU in critical condition. Despite intensive medical management, his condition deteriorated, and he passed away two days later.

## Discussion

Thyroid cancer accounts for 90% of endocrine malignancies, with PTC comprising approximately 80% of these cases [3]. Among its various subtypes is the hobnail variant, officially recognized in the 2017 WHO classification of tumors of endocrine organs [5] as a rare and aggressive form. This variant is characterized by severe histopathological features and is often resistant to radioiodine therapy, leading to a higher mortality rate compared to the classical variant of PTC [6]. Kakudo et al. were the first to identify this variant in 2004 as a poorly differentiated form of thyroid cancer, and its aggressive behavior was later confirmed in a Mayo Clinic case series published in 2010 [5,7,8]. The hobnail variant predominantly affects women and carries a higher risk of extrathyroidal extension, vascular and lymph node invasion, and frequently metastasizes to the lungs and bones [9].

The HPTC, which accounts for less than 1% of all PTC cases, is characterized by a distinctive cellular architecture, marked by a hobnail appearance of the cells and a micropapillary growth pattern [4]. To meet the diagnostic criteria for HPTC, at least 30% of the tumor cells must exhibit these features [9,10]. Even when these characteristics are less prominent, the diagnostic report should still note the presence of hobnail-micropapillary features. Recent studies have shown that tumors with ≥30% hobnail morphology are more likely to develop lymph node metastases compared to those with <30% [9].

Additionally, there is a strong association between HPTC and specific genetic mutations. The BRAF V600E mutation, in particular, has been linked to a higher risk of recurrence and reduced overall survival [10]. Other mutations, including TP53 and TERT, have also been identified in these tumors [11]. These genetic alterations significantly contribute to the aggressive nature of the disease but also open the door to potential targeted treatments, such as BRAF inhibitors, for patients harboring these mutations.

HPTC is typically diagnosed at an advanced stage and often presents as a rapidly enlarging neck mass with early metastatic potential [12]. In some cases, including ours, patients may present with symptoms such as dyspnea due to compression of adjacent structures. Given the limited number of cases and studies, understanding the natural history, treatment course, and long-term outcomes of HPTC remains a challenge.

In our case, clinical and pathological findings were strongly indicative of HPTC. Histological examination revealed papillae and micropapillae structures, nuclear inversion, and other aggressive cytologic features. However, it is important to note that definitive diagnosis remains limited in the absence of a surgical specimen. While the biopsy findings were consistent with HPTC, confirmation through surgical assessment would have been ideal.

## Conclusions

HPTC remains a rare and aggressive form of papillary thyroid cancer, presenting significant clinical challenges. This case highlights the importance of a multidisciplinary approach in the management of aggressive thyroid malignancies, particularly in complex situations where surgery is not a viable option. Further research is essential to better understand the clinical progression and histopathological features of HPTC, which are critical for guiding optimal treatment strategies and improving patient outcomes. It is also important to note that, in this case, the patient’s death was unrelated to cancer progression.
